# Regulation of microRNA-33, SREBP and ABCA1 genes in a mouse model of high cholesterol

**DOI:** 10.5194/aab-64-103-2021

**Published:** 2021-03-19

**Authors:** Xianglun Zhang, Hongbo Zhao, Qingkai Sheng, Xiaomu Liu, Wei You, Haichao Lin, Guifen Liu

**Affiliations:** 1 Institute of Animal Science and Veterinary Medicine, Shandong Academy of Agricultural Sciences, Jinan, China; 2 Shandong Key Lab of Animal Disease Control and Breeding, Jinan, China

## Abstract

MicroRNAs are short non-coding RNAs that regulate gene expression. Several microRNAs, useful for coronary artery disease assessment, have previously been identified. MicroRNA-33 is located within SREBP introns and controls cholesterol homeostasis. In order to find the possibility of microRNA-33 as a potential biomarker in high cholesterol disease, we developed a mouse model for coronary heart disease by feeding mice with a high-fat diet. The expression differences of microRNA-33, SREBP and ABCA1 genes in the liver, muscle, and lipid tissues were compared between a high-cholesterol group and control group in mice. The results showed that ABCA1 was up-regulated by high cholesterol conditions in liver, muscle and lipid tissues. SREBP1C was up-regulated by high cholesterol conditions in the liver and lipid tissues and down-regulated by high cholesterol conditions in the muscle tissue. MicroRNA-33 and SREBP2 were down-regulated by high cholesterol conditions in the liver and muscle tissues and up-regulated by high cholesterol conditions in the lipid tissue. Our study suggests that antisense therapeutic targeting of microRNA-33 may be a potential biomarker for cardiovascular disease.

## 1 Introduction

Cholesterol plays key role in many physiological process, and aberrant cholesterol content has been linked to disease, including coronary atherosclerosis and other diseases (Maxfield and Tabas, 2005; Moller and Kaufman, 2005). The classical transcription regulators, microRNAs, are important modulators of numerous cellular processes that impact disease (Williams et al., 2009). MicroRNAs, such as microRNA-126 (Fujii et al., 2016), microRNA-144 (Chen et al., 2018), and microRNA-33 (Kim et al., 2017), are a class of endogenous small, non-coding RNAs that bind to specific mRNAs to either inhibit their translation or promote their degradation (Lee et al., 1993; Reinhart et al., 2000; van Rooij and Olson, 2007). Circulating microRNAs are present within body fluids in a stable, cell-free form, and differing microRNA expression levels can be identified in various stages of coronary artery disease (CAD). Studies have shown a significant association between increased levels of plasma microRNA-33 and coronary artery disease, and therefore, microRNA-33 could be a useful biomarker for assessing CAD (Alrob et al., 2017; Gharipour and Sadeghi, 2013; Ono et al., 2015; Reddy et al., 2019; Rottiers and Naar, 2012; Rottiers et al., 2011).

MicroRNA-33a and microRNA-33b (the latter being present in primates but absent in rodents and lower species) are located within the introns of the sterol regulatory element binding protein (SREBP)-encoding genes. The SREBP cooperates with the proteins to control cholesterol homeostasis, fatty acid levels and expression of genes related to fat metabolism (Brown and Goldstein, 1997; Horie et al., 2010). SREBP-1c and SREBP-2 are two members of the SREBP family; there are differences in lengths. SREBP-1 regulated the gene expression which related to fatty acid synthesis. SREBP-2 regulated the gene expression which related to cholesterol uptake and synthesis (Moon et al., 2001).

Cholesterol plays a key role in many physiological processes. In lipid homeostasis, it has been linked to a number of diseases, including atherosclerosis (Najafi-Shoushtari et al., 2010). Cholesterol homeostasis in the liver is tightly controlled by pathways that regulate sterol synthesis and uptake from the plasma, which is mediated by SREBP and cholesterol catabolism. MicroRNA-33, encoded in an SREBP intron, targets the ATP-binding cassette transporter A1 (ABCA1) (an integration member protein) (Horie et al., 2010). ABCA1 can regulate high-density lipoprotein (HDL) synthesis and reverse cholesterol transport (Mao et al., 2014; Singaraja et al., 2002), implying that enhanced ABCA1 activity may protect against high cholesterol.

The microRNA-33, SREBP and ABCA1 genes have important role in cholesterol synthesis. Herein, we developed a mouse model of high cholesterol to investigate the functions of microRNA-33 and its related genes in mice.

## 2 Materials and methods

### 2.1 Animal experiment protocols

The animals used in this study were reared and sacrificed in compliance with national regulations for the humane care and use of animals in research (China Administration Rule of Laboratory Animals, Operating Procedure of Cattle Slaughtering GB/T 19477-2004). A total of 36 male C57BL mice weighing 18–20 g and aged 6 weeks were separated into two groups: one group was given water ad libitum and fed a high-fat diet containing 80 % ordinary feed, 10 % lard, 6 % egg yolk powder, 4 % cholesterol and 0.5 % sodium deoxycholate; another group was given water ad libitum and fed a standard laboratory chow diet for 30 d. On day 30, these mice were fasted for 12 h, weighed and anesthetized. Blood was collected from the mice to determine the serum levels of total cholesterol (TC), total triglyceride (TG), high-density lipoprotein (HDL) and low-density lipoprotein (LDL). Liver and muscle tissue were cut into small pieces and frozen in liquid nitrogen until the next step to detect gene expression.

### 2.2 RNA extraction

Total RNA was extracted using TRIzol Reagent (Life Technologies, Inc., Gaithersburg, MD, USA) in accordance with the manufacturer's instructions and stored at -80 ${}^{\circ }$C. The purity and integrity of RNA was evaluated via electrophoresis, ethidium bromide staining, and optical density (OD) absorption ratios OD260 / OD280 and rRNA (28S / 18S).

### 2.3 Quantitative real-time PCR

Expression of the selected genes was analyzed using the Fast SYBR^®^ Green Master Mix Bulk Pack (4385614; Invitrogen, USA). The following primers were used for quantitative reverse-transcription PCR (qRT-PCR): ABCA1, sense, 5${}^{\prime }$-CATTTCGAAGGAGACAAACATGTCA-3${}^{\prime }$ and antisense, 5${}^{\prime }$-CATGGCTTTATTCGGAAAGTGGCAC-3${}^{\prime }$; SREBP1C sense, 5${}^{\prime }$-GGCTGTTGTCATCCATAAGC-3${}^{\prime }$ and antisense 5${}^{\prime }$-AGGAAGAAACATGTCAAGAA-3${}^{\prime }$; SREBP2, sense 5${}^{\prime }$-TTCCCTTGTGTTGACCACGC-3${}^{\prime }$ and antisense 5${}^{\prime }$-TGTGGTCAGAATGGTCCCGT-3${}^{\prime }$; and β-actin, sense 5${}^{\prime }$-TCTCCACCTTCCAGCAGATGT-3${}^{\prime }$ and antisense 5${}^{\prime }$-AGCTCAGTAACAGTCCGCCTAGA-3${}^{\prime }$ (Beijing Dingguo Changsheng Biotechnology Co. Ltd.). Three replicates were used for each experiment. Relative mRNA expression levels were measured using the comparative Ct method (ΔΔCt) with β-actin as the internal control. The microRNA-33-3p sense sequence was CGCGCAATGTTTCCACAGTG, and the antisense sequence was AGTGCAGGGTCCGAGGTATT. The microRNA-33-3p-RT sequence was GTCGTATCCAGTGCAGGGTCCGAGGTATTCGCACTGGATACGACGTGATG.

**Figure 1:**
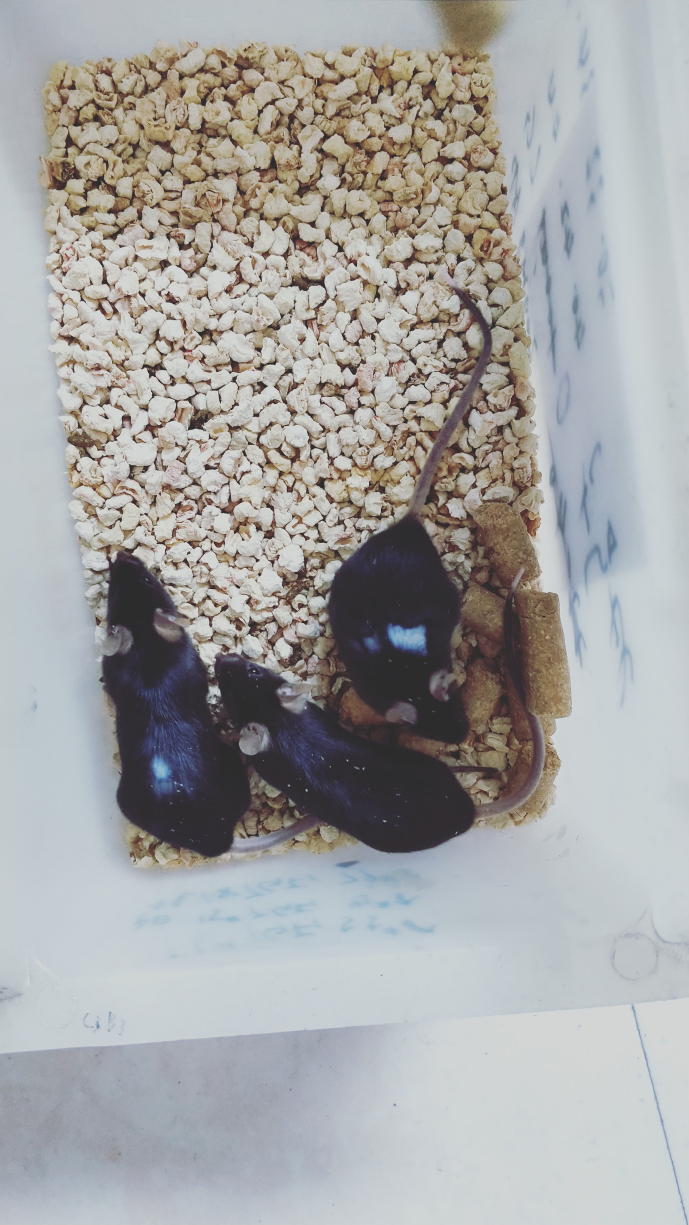
Mice in the experimental group.

**Figure 2:**
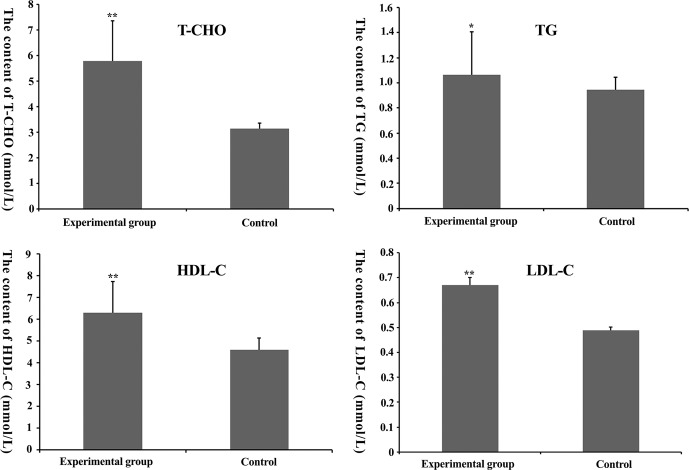
Total cholesterol (TC), total triglyceride (TG), high-density lipoprotein-cholesterol (HDL-C) and low-density lipoprotein-cholesterol (LDL-C) content in mouse serum. ${}^{*}$ denotes P<0.05. ${}^{**}$ denotes P<0.01.

**Figure 3:**
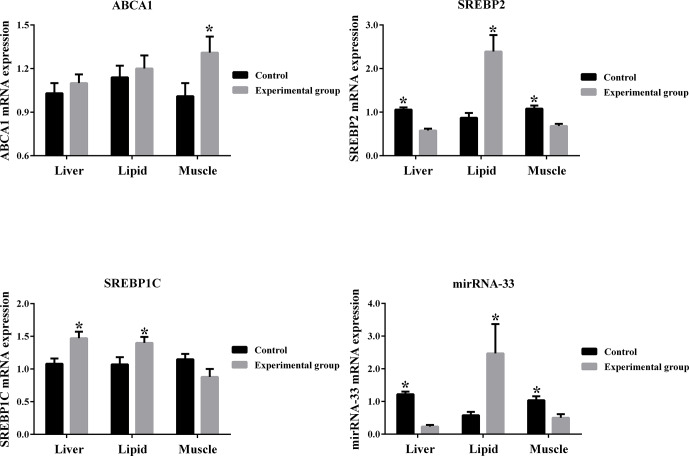
Gene expression in different mouse tissues between the control and experimental groups.

**Table 1:** The correlation between miRNA-33 and TG, TC, HDL-C and LDL-C.

		TC	TG	LDL-C	HDL-C
Liver miRNA-33	Pearson correlation	-0.690	-0.828	-0.706	-0.844
	Significance	0.040	0.006	0.034	0.004
Lipid miRNA-33	Pearson correlation	0.573	0.797	0.708	0.540
	Significance	0.107	0.010	0.033	0.134
Muscle miRNA-33	Pearson correlation	-0.498	-0.542	-0.528	-0.650
	Significance	0.070	0.045	0.052	0.012

### 2.4 Blood lipid analysis

TC, TG, HDL-C and LDL-C levels were measured in the plasma using detection kits specific to each cholesterol type (TC kit, TG kit, HDL-C kit and LDL-C kit; Nanjing Jiancheng Biology Institute, Nanjing, China).

### 2.5 Statistical analysis

SPSS software version 20.0 was used to perform statistical analysis. Data differences in gene expression among multiple groups were calculated using an analysis of variance, followed by a Bonferroni test. Comparisons between two groups were analyzed using the independent-sample t test. The Pearson correlation was used to analyze the correlation between genes and blood indexes.

## 3 Results

### 3.1 Animal experiment

After the experiment, mouse appearance was observed. From Fig. 1, we find that most of the mice in the experimental group lost their hair more or less by being fed a high cholesterol diet. The percent value of hair loss can be as high as 60 %.

### 3.2 TC, TG, HDL-C and LDL-C content in serum of mice

From Fig. 2, we find that T-C, TG, HDL-C and LDL-C content in the experimental group was higher than that in the control group (P<0.05), suggesting that the feasibility of the model was founded on nutrition.

### 3.3 Difference in gene expression in different tissues of mice between experimental group and control group

From Fig. 3 it can be seen that ABCA1 is up-regulated by high cholesterol conditions in the liver, muscle and lipid tissues of mice. SREBP1C is up-regulated by high cholesterol conditions in the liver and lipid tissues and down-regulated by high cholesterol conditions in the muscle tissue. Additionally, microRNA-33 and SREBP2 are down-regulated by high cholesterol conditions in the liver and muscle tissues and up-regulated by high cholesterol conditions in the lipid tissue.

### 3.4 Correlation between miRNA-33 and TG, TC, HDL-C and LDL-C

From Table 1 we observe a significant negative correlation between microRNA-33 and TC, TG, HDL-C and LDL-C in the liver and between microRNA-33 and TG, HDL-C in muscle tissues. However, there are significant positive correlations between microRNA-33 and TG as well as between microRNA-33 and LDL-C in the lipid tissue.

## 4 Discussion

Altered cholesterol homeostasis is involved in the pathogenesis of cicatricial alopecia; the mutation of cholesterol transporter is associated with congenital hypertrichosis (Palmer et al., 2020). The control of cellular cholesterol in the hair follicle and the potential impact on the hair cycle may identify novel target for regulating hair growth and the treatment of hair disorders linked to disordered sterol homeostasis or sterol-sensitive signaling pathways (DeStefano et al., 2014; Evers et al., 2010). To the best of our knowledge, this is one of the few studies wherein diet appeared to affect the hair loss of mice. In the current study, the ApoE${}^{-/-}$ mice (fed diets rich in cholesterol and fat) showed skin inflammation, hair discoloration and hair loss (Bedja et al., 2018). It is not clear whether such hair loss will appear in humans in the future, but this must be tested clinically. Although this result is rarely discussed in the literature, we believe that it is an important theory to be tested in future research.

MicroRNA-33 has been shown to be up-regulated by low sterol conditions, including lipid deprivation and statin treatment, and down-regulated by lipid loading (Marquart et al., 2010; Najafi-Shoushtari et al., 2010; Rayner et al., 2010). MicroRNA-33 levels were positively correlated with the TC and LDL-C levels but negatively correlated with the HDL-C levels in blood (Chen et al., 2016). Long-term anti-microRNA-33 therapy significantly reduces the progression of atherosclerosis and improves HDL functionality (Rotllan et al., 2013). Further, RT-PCR analysis of miRNA-33 in the blood plasma of patients with CAD showed a 2.9-fold increase compared to that in the blood plasma of the control group, supporting the use of microRNA-33 as a potential biomarker for CAD (Reddy et al., 2019). Additionally, hepatic microRNA-33 levels show a 1.5–2.5-fold increase under physiological conditions that alter SREBP-2 expression (Tarling et al., 2015).

Consistent with the results of studies demonstrating the coordinated regulation of SREBP2 and microRNA-33 (Elizabeth et al., 2015), our results indicate that these two genes have the same expression trends in the three tissues when the high cholesterol diets also increase/decrease the microRNA-33 expression in parallel with the alteration in SREBP-2 mRNA levels in the tissues. We found that microRNA-33 and SREBP2 were down-regulated by high cholesterol conditions in the liver and muscle tissues and up-regulated by high cholesterol conditions in the lipid tissue.

In summary, our results indicate that the key regulators of cholesterogenic and lipogenic genes combine with microRNA-33 to govern intracellular cholesterol levels and cholesterol homeostasis in mice. We propose antisense therapeutic targeting of microRNA-33 as a potential strategy for the management of cardiovascular disease.

## 5 Conclusion

Our study suggests that antisense therapeutic targeting of microRNA-33 may be a potential biomarker for cardiovascular disease.

## Data Availability

The data are available from the corresponding author upon request.

## Code availability

SPSS software (version 20.0) is available at (IBM, 2021).
